# A Dual-Optimization Fault Diagnosis Method for Rolling Bearings Based on Hierarchical Slope Entropy and SVM Synergized with Shark Optimization Algorithm

**DOI:** 10.3390/s23125630

**Published:** 2023-06-16

**Authors:** Yuxing Li, Bingzhao Tang, Bo Huang, Xiaohui Xue

**Affiliations:** 1 School of Automation and Information Engineering, Xi’an University of Technology, Xi’an 710048, China; 2210321205@stu.xaut.edu.cn (B.T.);; 2 Shaanxi Key Laboratory of Complex System Control and Intelligent Information Processing, Xi’an University of Technology, Xi’an 710048, China

**Keywords:** fault diagnosis, hierarchical slope entropy, white shark optimizer, optimized support vector machine, bearing signals

## Abstract

Slope entropy (SlopEn) has been widely applied in fault diagnosis and has exhibited excellent performance, while SlopEn suffers from the problem of threshold selection. Aiming to further enhance the identifying capability of SlopEn in fault diagnosis, on the basis of SlopEn, the concept of hierarchy is introduced, and a new complexity feature, namely hierarchical slope entropy (HSlopEn), is proposed. Meanwhile, to address the problems of the threshold selection of HSlopEn and a support vector machine (SVM), the white shark optimizer (WSO) is applied to optimize both HSlopEn and an SVM, and WSO-HSlopEn and WSO-SVM are proposed, respectively. Then, a dual-optimization fault diagnosis method for rolling bearings based on WSO-HSlopEn and WSO-SVM is put forward. We conducted measured experiments on single- and multi-feature scenarios, and the experimental results demonstrated that whether single-feature or multi-feature, the WSO-HSlopEn and WSO-SVM fault diagnosis method has the highest recognition rate compared to other hierarchical entropies; moreover, under multi-features, the recognition rates are all higher than 97.5%, and the more features we select, the better the recognition effect. When five nodes are selected, the highest recognition rate reaches 100%.

## 1. Introduction

Rolling bearings, as a key component in rotating machinery, serve a very significant role in modern industry. However, because of the increasingly sophisticated and complex structure of bearings and their common use in harsh working environments, rolling bearings are very prone to failures, which can lead to economic losses and even endanger personal safety [1,2,3]. Therefore, aiming to ensure the normal work of rotating machinery and reduce maintenance costs, it is of great importance to carry out fault diagnoses of rolling bearings [4,5,6].

Since bearing vibration signals contain rich state information about the bearing during operation, a vibration analysis method is broadly applied to rolling bearing faults [7,8]. In general, the method mainly consists of two steps: feature extraction and fault classification, in which valid feature extraction is crucial for accurate fault diagnosis. As the bearing vibration signal has nonlinear dynamic characteristics, traditional feature extraction methods based on Fourier transform and statistical analysis only characterize features from the time domain or frequency domain, and they cannot detect potential faults through changes in the complexity of the system to achieve effective and accurate extractions of fault features [9,10].

In recent years, nonlinear dynamic methods, such as sample entropy (SE) [11], permutation entropy (PE) [12], and dispersion entropy (DE) [13], have been widely used in the feature extraction of bearing signals and have presented superior performance. Han et al., used SE to extract bearing fault feature information effectively [14], but SE calculation is complicated and not suitable for real-time monitoring. As PE has the strengths of fast calculation and good stability, Xue et al., proposed a bearing fault diagnosis method based on PE and further improved the effectiveness of fault diagnosis [15]. While PE does not consider amplitude information, Dhandapani et al., applied DE to the feature extraction osf rolling bearing faults and considered the amplitude information of bearing signals [16]. Unlike the above entropies, slope entropy (SlopEn) is a new entropy estimator proposed based on symbolic patterns and magnitude information [17] and has been applied in the underwater acoustic field and medical field many times [18,19,20,21]. In 2022, SlopEn was introduced into the field of bearing fault diagnosis for the first time, and experimental results showed that, compared with PE and DE, SlopEn could better extract fault information [22]. However, all the above-mentioned entropy-based bearing fault diagnosis methods suffer from two defects: (i) the methods extract only the fault information of the low-frequency component for the bearing signal, and (ii) there is the problem of threshold selection for SlopEn, and the thresholds usually need to be optimized using an optimization algorithm.

Aiming at extracting the bearing fault information more comprehensively, some scholars have proposed fault diagnosis methods based on hierarchical entropy [23,24,25]. The authors of [26] proposed the concept of hierarchical permutation entropy (HPE) and employed it successfully for fault diagnosis. Moreover, Ref. [27] used hierarchical permutation entropy (HDE) to extract the fault information in both high- and low-frequency components. Diagnosis methods based on hierarchical entropy have proved that they can obtain diagnosis-related information of the whole frequency band and have strong noise resistance and stability; in addition, no scholars have introduced the concept of hierarchy to the SlopEn and used optimization algorithms to optimize the thresholds.

After feature extraction, the next step is fault classification. Commonly used fault classification methods mainly include k-nearest neighbor (KNN) [28], random forest (RF) [29], and the support vector machine (SVM) [30]. The SVM has been widely used in fault diagnosis because of its suitability for small sample classification and its simple structure [31]. Yet, since the parameter penalty factors and kernel functions of SVM models have an impact on fault diagnosis, some existing optimization algorithms have optimized the parameters of SVMs and improved the performance of fault classification, such as the genetic algorithm (GA) [32], particle swarm algorithm (PSO) [33], and whale optimization algorithm (WOA) [34]. Compared to common optimization algorithms, the white shark optimizer (WSO) is a new meta-heuristic optimization algorithm based on deep-sea foraging by great white sharks, proposed in 2022 for solving optimization problems on continuous search spaces [35]; in addition, the results of the basis function tests show that WSO is better than the common optimization algorithm in terms of optimization and has not yet been applied to optimize SVMs.

Based on the analysis above, a dual-optimization fault diagnosis method for rolling bearings is put forward, and the main novelties and contributions of this paper are presented as follows:

(1) To extract the fault information of bearing signals more comprehensively, on the basis of SlopEn, this paper adds the concept of hierarchy and is the first to propose hierarchical slope entropy (HSlopEn).
(2) Since the thresholds of HSlopEn have a relatively large impact on the entropy value and the selection of suitable parameters of an SVM is particularly important for the classification, this paper applies the WSO to optimize the parameters of HSlopEn and an SVM and proposes WSO-HSlopEn and WSO-SVM, respectively.
(3) Targeting the application of bearing fault diagnosis under different operating conditions, this paper proposes a dual-optimization fault diagnosis method for rolling bearings based on HSlopEn and an SVM synergized with the WSO.

The remaining parts of this paper are structured as follows. Section 2 presents the basic concepts of algorithms. Section 3 introduces the steps of the proposed fault diagnosis method. Section 4 carries out the single-feature and multi-feature extraction experiments for bearing signals, and Section 5 summarizes the conclusions of this study.

## 2. Methodology

### 2.1. Slope Entropy

**Figure 1:**
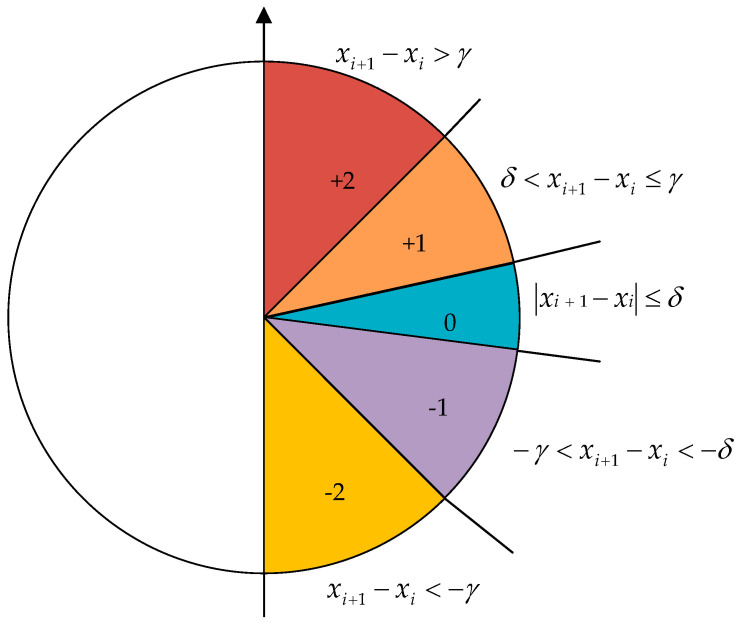
The symbol allocation of SlopEn.

Slope entropy (SlopEn) is an algorithm proposed in 2019 to calculate the complexity of time series. It is based on symbolic patterns and magnitude information. The main calculation steps are listed below:

(1) For a given time series $X=\left\{x_{1},x_{2},\cdots ,x_{N}\right\}$, according to the embedding dimension m, extract the subsequences:$X_{1}=\left\{x_{1},x_{2},\cdots ,x_{m}\right\}{,X}_{2}=\left\{x_{2},x_{3},\ldots ,x_{m-1}\right\},\ldots ,X_{k}=\left\{x_{k},x_{k+1},\ldots ,x_{N}\right\}$, of which k=N−m+1.
(2) Two threshold parameters, γ and δ, are used to delimit the symbolic patterns including (+2, +1, 0, −1, −2) between two consecutive samples $x_{i+1}-x_{i}$ of the subsequence $X_{1},X_{2},\cdots ,X_{k}$. When $|x_{i+1}-x_{i}|\leq \delta$, the symbol is 0; when $\delta <x_{i+1}-x_{i}<\gamma$, the symbol is +1; when $-\gamma <x_{i+1}-x_{i}<-\delta$, the symbol is −1; when $\gamma <x_{i+1}-x_{i}$, when symbol is +2; when $x_{i+1}-x_{i}<-\gamma$, the symbol is −2, where γ>δ>0. Figure 1 displays the symbol allocation of SlopEn.
(3) According to the symbol pattern of step (2), divide all the subsequences $X_{1},X_{2},\cdots ,X_{k}$, and obtain the symbol pattern sequences $Y_{1}=\left\{y_{1},y_{2},\ldots ,y_{m-1}\right\}{,Y}_{2}=\left\{y_{2},y_{3},\cdots ,y_{m}\right\}{,\ldots ,Y}_{k}=\left\{y_{k},y_{k+1},\cdots ,y_{N-1}\right\}$, where $y_{1}$ is obtained from $x_{2}-x_{1}$ through step (2), $y_{2}$ is obtained from $x_{3}-x_{2}$ through step (2), … , and $y_{N-1}$ is obtained from $x_{N}-x_{N-1}$ through step (2).
(4) The total number of types of symbol pattern sequences is recorded as $r=5^{m-1}$, and the number of occurrences of each symbol pattern sequence is $s_{1},s_{2},\cdots ,s_{r}$, respectively. Hence, the probability of each symbol pattern sequence appearing is $P_{1}=\frac{s_{1}}{r},P_{2}=\frac{s_{2}}{r},\ldots ,P_{r}=\frac{s_{r}}{r}$, respectively.
(5) On the basis of Shannon information entropy, the definition formula of SlopEn is
  (1) $$SlopEn(X,m,\gamma ,\delta )=-\sum_{i=1}^{r}P_{r}lnP_{r}$$

### 2.2. Hierarchical Slope Entropy

Since SlopEn only considers the low-frequency components of the time series, aiming to describe the time series more comprehensively, on the basis of SlopEn and combined with the concept of hierarchy, this paper proposes a new complexity feature, namely hierarchical slope entropy (HSlopEn). The specific process of HSlopEn is as follows:

(1) First, given a time series $X=\left\{x\left(i\right),i=0,1,\cdots ,N,N=2^{n}\right\}$ of length N, define an average operator $Q_{0}\left(x\right)$ and a difference operator $Q_{1}\left(x\right)$, which can be expressed as

(2) $$Q_{0}=\frac{x\left(2j\right)+x\left(2j+1\right)}{2},j=0,1,2,\cdots ,2^{n-1}$$

(3) $$Q_{1}=\frac{x\left(2j\right)-x\left(2j+1\right)}{2},j=0,1,2,\cdots ,2^{n-1}$$

where the $Q_{0}\left(x\right)$ and $Q_{1}\left(x\right)$ operators are the low-frequency part and high-frequency part, respectively, of the original given time series after hierarchical decomposition and n is a positive integer.

(2) The operators $Q_{j}(j=0\;or\;1)$ in matrix form is defined as
  (4) $$Q_{j}={\left[\begin{array}{ccccccc} \frac{1}{2} & {\left(\frac{-1}{2}\right)}^{j} & 0 & 0 & \cdots & 0 & 0\\ 0 & 0 & \frac{1}{2} & {\left(\frac{-1}{2}\right)}^{j} & \cdots & 0 & 0\\ 0 & 0 & 0 & 0 & \cdots & \frac{1}{2} & {\left(\frac{-1}{2}\right)}^{j} \end{array}\right]}_{2^{n-1}\times 2^{n}}$$
(3) The l-dimension vectors $\left[u_{1},u_{2},\ldots ,u_{l}\right]\in \{0,1\}$(lϵN) are constructed, and an integer e can be expressed:

(5) $$e=\sum_{j=1}^{l}u_{j}2^{l-j}$$

where, for a positive e, there is a unique set of l-dimension vectors $\left[u_{1},u_{2},\ldots ,u_{l}\right]\in \{0,1\}$ and the positive integer e represents the sequence number of the node at each layer, where $0⩽e⩽2^{n-1}$.

(4) The hierarchical decomposition of a given time series X yields a hierarchical component corresponding to the node e at the Kth level, defined as
  (6) $$X_{K,e}=Q_{u_{l}}*Q_{u_{l-1}}*\cdots *Q_{u_{1}}\left(x\right)*X$$
(5) By calculating the SlopEn of nodes on different layers, the HSlopEn can be expressed as

(7) $$HSlopEn\left(X,m,K,\gamma ,\delta \right)=SlopEn\left(X_{n,e},m,\gamma ,\delta \right)$$

where K is the number of layers of decomposition, γ and δ are the two thresholds of SlopEn, and m is the embedding dimension.

**Figure 2:**
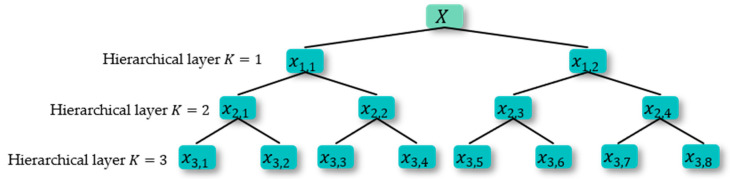
Hierarchical decomposition structure diagram when K = 3.

As displayed in Figure 2, the hierarchical decomposition structure diagram when K = 3 is shown. SlopEn is calculated on each node after the hierarchical decomposition. 

In Figure 2, X indicates the original time series, $x_{1,1}$ is the first node of the first layer, $x_{2,1}$ is the first node of the second layer, and so on.

### 2.3. Analysis of the Parameters for HSlopEn

The main parameters of HSlopEn include the number of decomposition layers K, embedding dimension m, two threshold parameters γ and δ*,* and time delay d. First, the number of decomposition layers K determines the number of nodes in the hierarchical decomposition. When the number of decomposition layers is too large, the number of nodes decomposed is too large, resulting in a large number of calculations for SlopEn values of all nodes; when the value is too small, resulting in a small number of decomposed nodes, there are insufficient frequency bands for the given time series. Referring to other references, the default number of decomposition layers K is 3 in this paper. Then, the embedding dimension m is used to extract the subsequence of a given time series. If it is too small, it is difficult to determine the dynamic changes of the time series; if it is too large, it is difficult to capture the subtle changes in the time series. After that, the two threshold parameters γ and δ are used to divide the symbol pattern of a given subsequence, which affects the change in entropy value. Lastly, the default time delay d is 1, as important information about frequency may be lost at that time if d>1. The effect of embedding dimension and thresholds on the performance of the HSlopEn is investigated below by analyzing the noisy signals.

**Figure 3:**
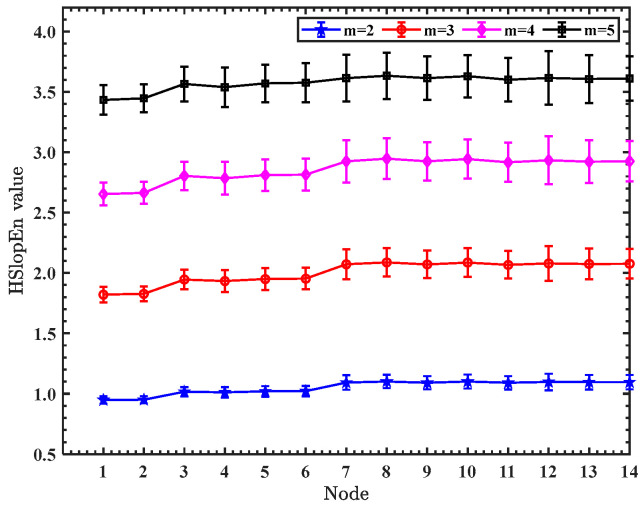
The mean and standard deviation (SD) of HSlopEn values for different embedding dimensions in every node.

To investigate the effect of embedding dimension on the entropy value of hierarchical slope entropy, 50 sets of white Gaussian noise (WGN) of signal length 2048 are used, with the embedding dimension m varying from 2 to 5 and the two threshold parameters γ and δ defaulting to 0.1 and 0.001, respectively. Figure 3 shows the mean and standard deviation (SD) of the HSlopEn values for different embedding dimensions in every node.

As shown in Figure 3, as the embedding dimension m becomes larger, the entropy value of the HSlopEn also becomes larger, but the entropy value of each node for HSlopEn is close to others at different embedding dimensions, and the difference between the mean and SD is small, which indicates that the change in the embedding dimension affects the size of the entropy value, but the stability of the HSlopEn hardly changes. The embedding dimension m is set to 3 in this paper.

**Figure 4:**
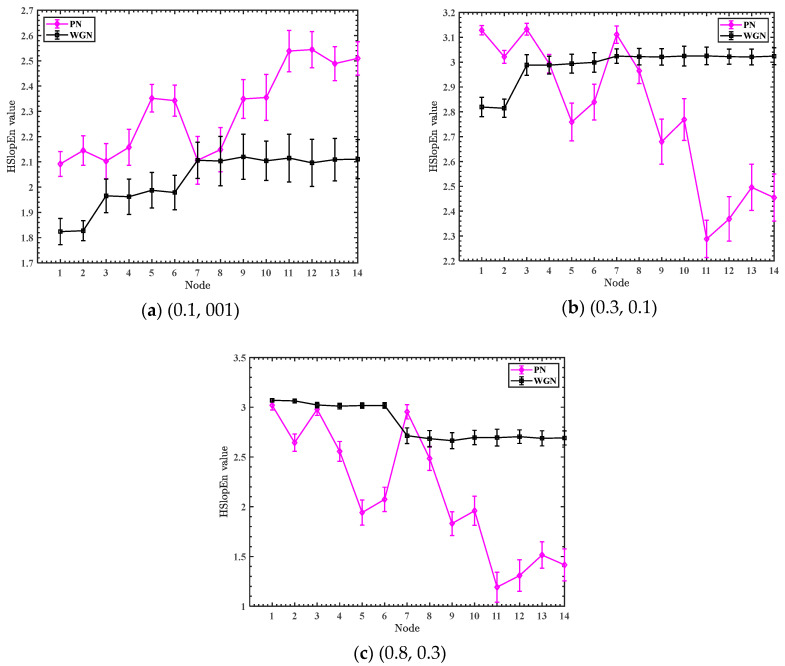
The mean and SD of HSlopEn values for three sets of thresholds in every node.

In addition, to further study the effect of thresholds γ and δ on the entropy of the HSlopEn, 50 independent pink noise (PN) and WGN signals are selected, where each noise is sampled at 2048 Hz and the embedding dimension m is 3. The three sets of thresholds (γ,δ) for HSlopEn are manually set, which are (0.1, 001), (0.3, 0.1), and (0.8, 0.3), and the mean and standard deviation (SD) of the HSlopEn values for the three sets of thresholds in every node are displayed in Figure 4.

It can be seen in Figure 4 that, as the threshold changes, the entropy values of the two types of noise signals change; at the same time, the ability to discriminate between the noise signals is constantly changing, so the threshold has a significant effect on the entropy of the HSlopEn. The WSO is used in the paper to optimize the thresholds to avoid taking values based on artificial experience and further improve the fault diagnosis.

### 2.4. WSO-HSlopEn and WSO-SVM

Following the principle of the HSlopEn algorithm, the two threshold parameters γ and δ of the HSlopEn are used to divide the sign pattern of a given time sequence subsequence. Thus, the two threshold parameters have a great influence on the HSlopEn value. At the same time, the classification effect of the support vector machine (SVM) mainly depends on the selection of the penalty factor (C) and kernel function parameters (g), and it is generally difficult to take the values based on manual experience. Hence, the selection of an appropriate penalty factor and kernel function parameters is also particularly important for the classification and recognition accuracy of the SVM. 

**Figure 5:**
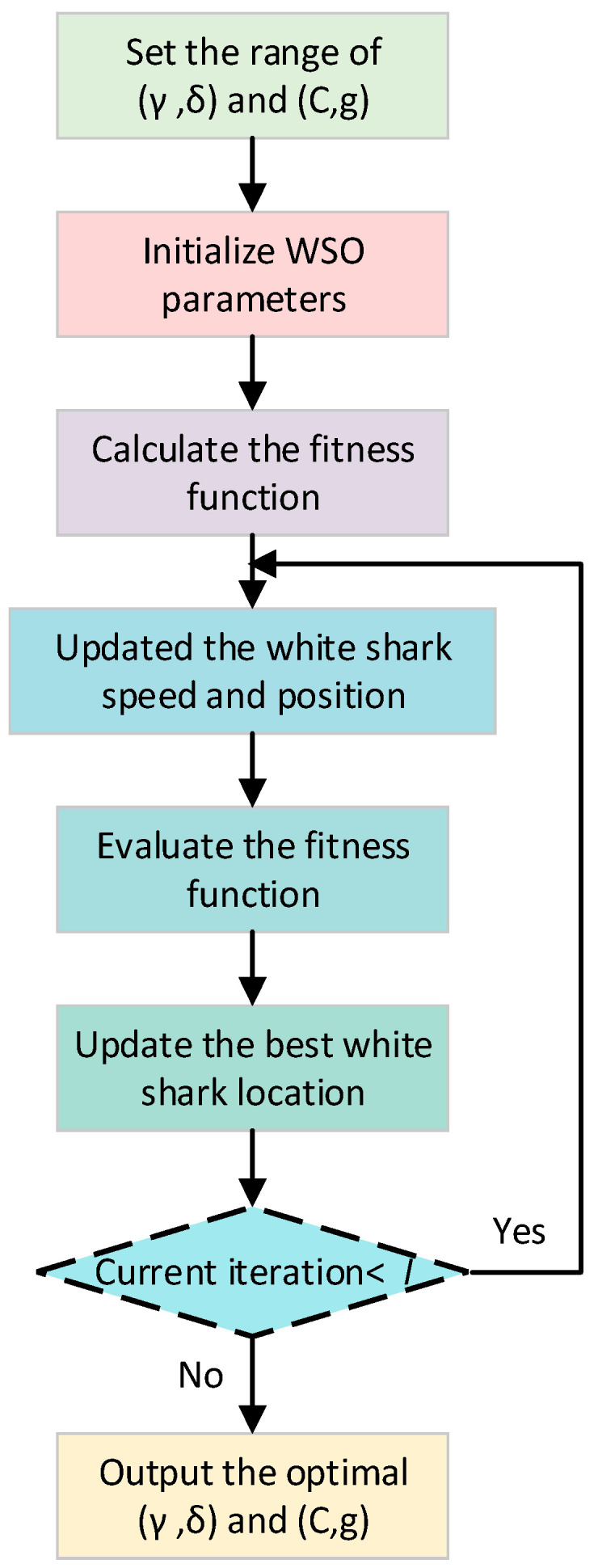
The main process of optimizing the parameters of the HSlopEn and SVM.

To enhance the performance of the fault diagnosis effect, in this paper, taking the recognition rate as the fitness function, the white shark optimizer (WSO) is used to optimize the parameters of HSlopEn and the SVM, and WSO-HSlopEn and WSO-SVM are proposed, respectively, where the WSO is a new meta-heuristic optimization algorithm based on deep-sea foraging by great white sharks, proposed in 2022 for solving optimization problems on continuous search spaces. The main process of optimizing the parameters of HSlopEn and the SVM is shown in Figure 5, and the specific process is as follows:

(1) Set the initial parameter ranges of HSlopEn (γ,δ) and the SVM (C,g).
(2) Initialize the WSO parameters, such as population size, number of iterations I, position, and speed of white sharks.
(3) Calculate the fitness function, and update the white sharks’ position and speed.
(4) Evaluate the fitness function, and update the optimal white shark position
(5) Update the position and speed of the white shark.
(6) Judge whether the current iteration number reaches the maximum iteration number. If so, return to update the speed and position of the white shark and repeat the above steps; otherwise, output the best-optimized parameters (γ,δ) and (C,g).

## 3. The Proposed Method for Fault Diagnosis of Rolling Bearing

**Figure 6:**
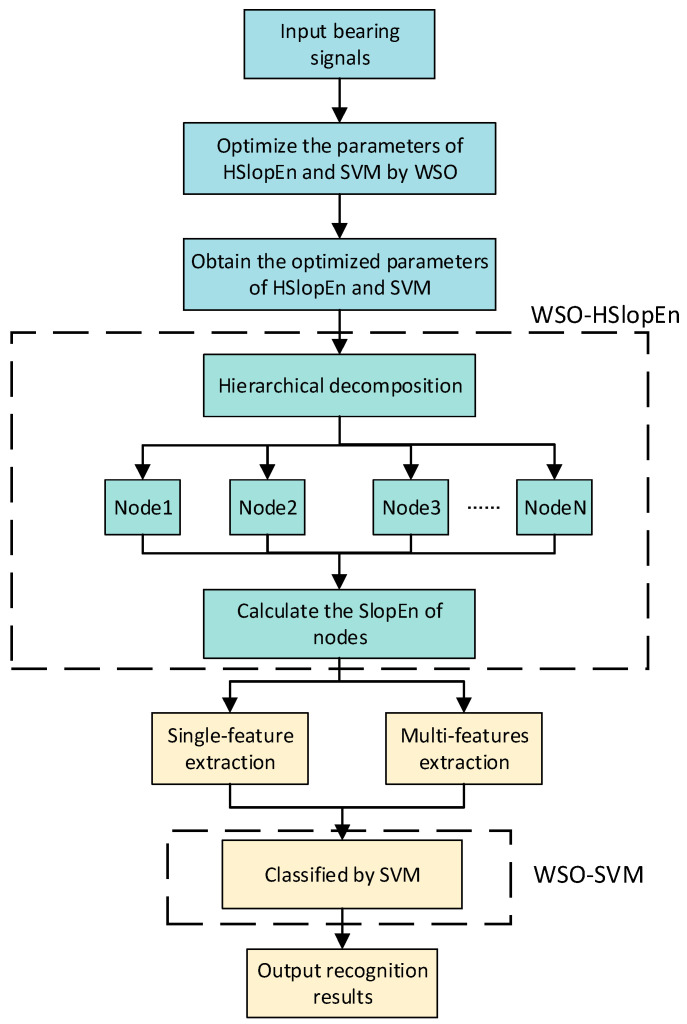
The flowchart of proposed dual-optimization fault diagnosis method for rolling bearings, based on WSO-HSlopEn and WSO-SVM.

Combining the concept of hierarchical structure, the new complexity feature HSlopEn is proposed, and the parameters of both HSlopEn and the SVM are optimized using the WSO algorithm, and WSO-HSlopEn and WSO-SVM are proposed, respectively. Then, a dual-optimization fault diagnosis method for rolling bearings based on WSO-HSlopEn and WSO-SVM is proposed. Figure 6 presents the flowchart of the proposed fault diagnosis method, and the method mainly includes the following steps:

(1) The different bearing signals are input. In this paper, each type of bearing signal has 100 samples with 1024 data points.
(2) The WSO algorithm is applied to optimize the parameters of HSlopEn (γ,δ) and the SVM (C,g) by taking the final recognition rate as the fitness function, and the optimized parameters are obtained. At the same time, other optimization algorithms, including SO, marine predator algorithm (MPA), and sparrow search algorithm (SSA), are used for comparison.
(3) Different types of bearing signals are decomposed into several layers, and the nodes are obtained. In this paper, bearing signals are decomposed into three layers.
(4) The nodes of WSO-HSlopEn are calculated, and then single-feature and multi-feature extraction experiments for bearing signals are carried out. Meanwhile, comparisons with some classical entropies, such as HFE, HPE, HSE, and HRDE, are conducted.
(5) WSO-SVM is applied to classify bearing signals, and the recognition results are output. In this paper, for each type, select 25 sample signals as training samples and 75 sample signals as test samples.

## 4. Experiments and Results

In this chapter, two comparative experiments are implemented to examine the effectiveness of the proposed method in fault diagnosis: (1) In optimizing both HSlopEn and SVM parameters using the WSO, we compare different optimization algorithms, including SSA, MPA, and SO. (2) In extracting the WSO-HSlopEn of nodes, we compare classical hierarchical entropy metrics, including HPE, HSE, HFE, and HRDE.

### 4.1. Fault Diagnosis of Rolling Bearing Signal

**Figure 7:**
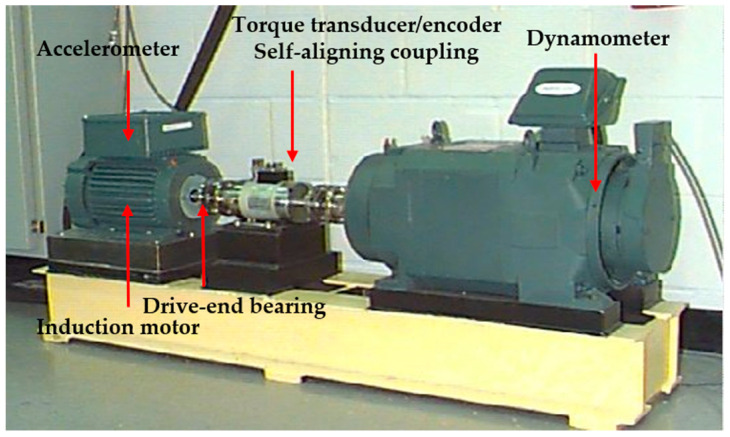
The schematic of test rig.

The dataset used in this section was derived from the Bearing Data Center of Case Western Reserve University [36], which is an internationally recognized standard dataset for fault diagnosis of rolling bearings. The schematic of the test rig (Cleveland, USA) is shown in Figure 7.

As shown in Figure 7, the test rig consisted of an induction motor, drive-end bearing, self-aligning coupling, and accelerometer dynamometer. An accelerometer was installed on the base of the motor, which was used to detect the vibration acceleration of the faulty bearing at a sampling frequency of 12 kHz. The dataset divided the fault data into four categories: normal data (NOR), ball faults (BFs), outer race faults (ORFs), and inner race faults (IRFs). Among them, BFs, ORFs, and IRFs were simulated faults with single-point damage as an electric spark. The damage diameters were divided into 0.007, 0.014, and 0.021 inches. At the same time, the processed faulty bearing was reloaded into the test motor, and the vibration acceleration signal data were recorded under the load working conditions of 0, 1, 2, and 3 horsepower.

**Figure 8:**
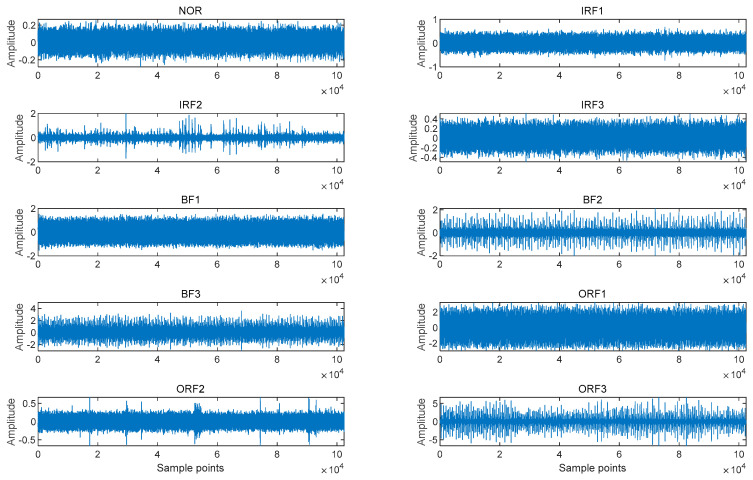
Time-domain waveforms for each state bearing signals.

In this section, bearing signals with ten conditions were collected from the drive-end bearings, including rolling bearings in normal condition and those with damage to the inner race, the outer race, and the ball element. Bearings with various damage diameters were considered under a speed of 1730 rpm with a load of 3 horsepower. Table 1 illustrates the fault diagnosis sample collection of bearing signals. Each fault signal was divided into three types according to the fault diameter. We sampled from point 1001, and each condition had 100 samples with 1024 sampling points. Time-domain waveforms for each state bearing signals are displayed in Figure 8.

### 4.2. Comparison of Different Optimization Algorithms

**Figure 9:**
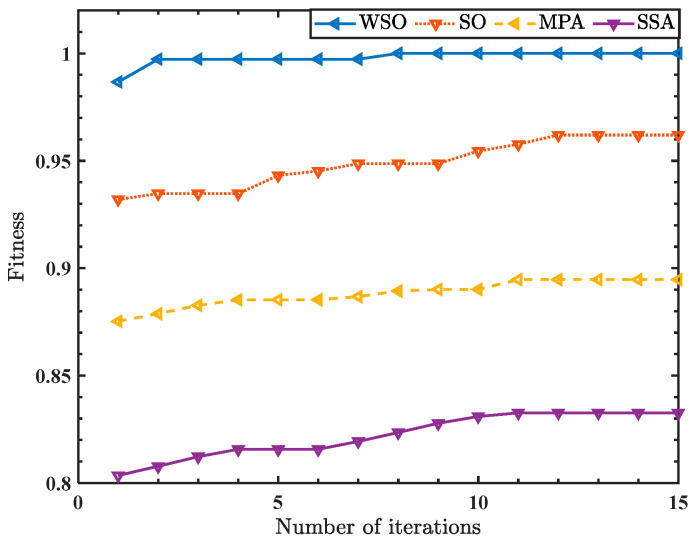
The fitness iteration curve of different optimization algorithms.

Designed to verify the performance advantages of the WSO in optimizing HSlopEn and the SVM, this section introduces different optimization algorithms to optimize the parameters of HSlopEn and the SVM, and compares recognition rates of single-feature and multi-feature extractions with those of other optimization algorithms [37,38,39]. In this experiment, 10 different bearing signal conditions were sampled from the 1001 point as the starting point, and 100 samples were selected. Each sample had 1024 data points. First, the parameters of HSlopEn were set as follows: hierarchical layer K=3, embedding dimension m=3, and threshold parameters γ and δ were adaptively determined using different optimization algorithms. HSlopEn with optimized parameters of bearing signals was extracted. Then, the sample set was divided into the training set and test set, and the select single feature or multi-features were input to optimize the SVM. The penalty factor and kernel function parameters of the SVM were also adaptively determined using the WSO algorithm. Figure 9 presents the fitness iteration curves of different optimization algorithms to optimize HSlopEn and the SVM. These are the fitness iteration curves of different optimization algorithms in the case of extracting five nodes.

It can be found in Figure 9 that, in the condition of extracting five nodes, the highest recognition rate of these ten types of bearing signals reached 100% using the WSO to optimize HSlopEn. At the same time, the convergence speed of the WSO was relatively faster than other optimization algorithms. In addition, the early convergence of the WSO is quick. Its fitness curve eventually converged to a bigger value. To further demonstrate the significant advantages of WSO, we calculated the recognition rate of bearing signals based on using different optimization algorithms to optimize HSlopEn and the SVM under the situation of extracting the single feature and multi-features. The recognition rates of HSlopEn for all single-feature nodes are shown in Table 2 and Table 3, and the highest recognition rates of HSlopEn for multi-features for the four types of optimization algorithms are shown in Table 4.

According to the recognition rate of different types of bearing signals, we can find that no matter how many features are extracted, the advantages of the WSO algorithm are obvious. In the case of extracting a single feature, the recognition rate of the WSO reaches 79.33% on node 6, which is much higher than that of other optimization algorithms. Under the circumstances of extracting multi-features, as the number of selected nodes increases, the recognition rate also improves. When we select five features, it realizes the correct identification of all samples. The recognition rate of other optimization algorithms, including SO, MPA, and SSA, is, respectively, 3.8%, 10.53%, and 17.73% lower than that of WSO. Above all, we prove that using the WSO to optimize HSlopEn and the SVM is feasible. Therefore, in this paper, the WSO is used to optimize HSlopEn and SVM parameters.

### 4.3. Comparison of Different Hierarchical Entropies

**Figure 10:**
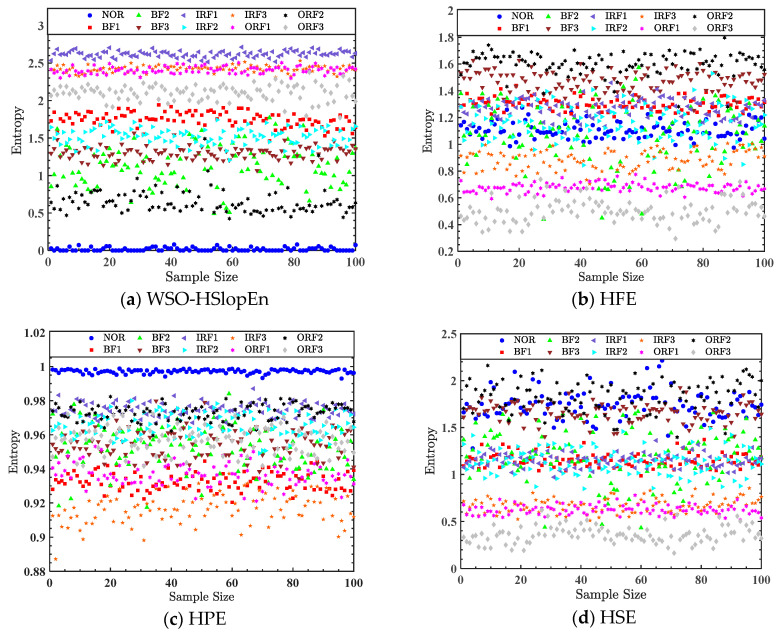

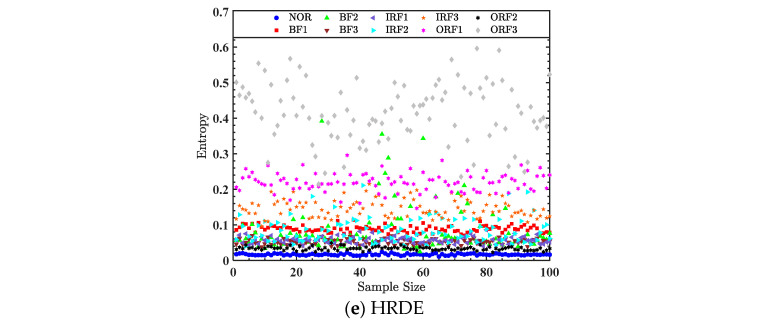
Entropy distribution of optimal node for single-feature extraction of bearing signals.

Aiming to demonstrate the superiority of WSO-HSlopEn in fault diagnosis, we compared it to other classical hierarchical entropies, including HSE, HFE, HPE, and HRDE. The single-feature approach was first used to extract the fault feature and compare it with HFE, HSE, HPE, and HRDE. The parameters of HSlopEn were as follows: hierarchical layer K=3, embedding dimension m=3, time delay d=1, and threshold parameter γ and δ were adaptively determined using the WSO algorithm. For a fair comparison, the parameter settings of other hierarchical entropies were the same as those in the HSlopEn method. Among them, the similarity tolerances of HSE and HFE were set as r=0.2, and the category number of HRDE was set as c=3. The entropy distributions of an optimal node for the single-feature extraction of bearing signals are shown in Figure 10.

Figure 10a presents that there was no aliasing phenomenon between the features of the three fault types of NOR, ORF3, and IRF1 and other fault types in the entropy distribution of WSO-HSlopEn. ORF2 only had a few samples of entropy close to BF2, and the entropy values of the samples of the other fault types show a severe overlap. Based on the single-feature extraction shown in Figure 10b–e, compared with other hierarchical entropies, WSO-HSlopEn is not as serious as the aliasing of the distributions of other hierarchical entropies. The entropy values of several types of samples in the ten conditions of bearing signals are quite different from the other types. Relatively speaking, the distance between the various types of samples in the distribution of the entropy of WSO-HSlopEn is relatively large.

After using the WSO-HSlopEn as the fault feature of the bearing signal, the bearing fault diagnosis sample set was divided into a training set and a test set, and the training set was input into the WSO-SVM to train the model, and then the test set was input into the model to finish the fault diagnosis of bearings. The Gaussian kernel function was selected as the kernel function of the SVM. The penalty factor and kernel function parameters of the SVM were also adaptively determined by the WSO algorithm. Recognition rates of single features for the five types of hierarchical entropies are displayed in Table 5 and Table 6.

Table 5 and Table 6 illustrate that, when using WSO-HSlopEn, the recognition rate of node 6 was the highest, which was 79.33%. Compared with other hierarchical entropies, under each node, the recognition rate based on WSO-HSlopEn was always the highest, which shows the effectiveness of WSO-HSlopEn as a fault diagnosis feature of bearing signals.

**Figure 11:**
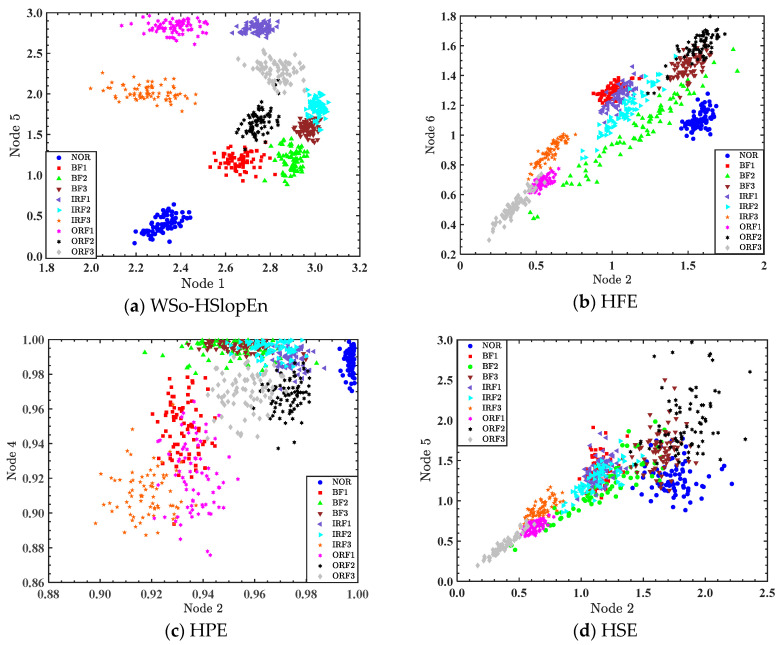

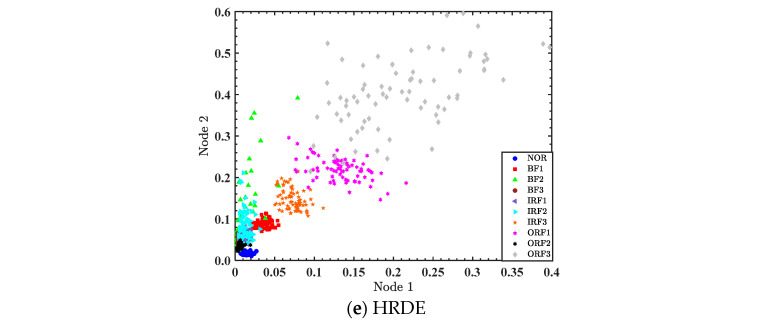
Double-features distribution of ten types of bearing signals.

Through observation, when single-feature extraction is used to extract the fault feature, there is still overlap between the features of different conditions of the bearing signals. Furthermore, the recognition rate of the best node was low, and there were many misclassified samples based on single-feature extraction. Aiming to further improve the recognition rate of different conditions of the bearing signals, double features were used to extract the bearing signals. All parameters used in the experiments were the same as those listed in the single-feature extraction. The entropy distribution on the optimal node for double-feature extractions of bearing signals is shown in Figure 11, where the abscissa and ordinate are the entropy values of the two nodes, respectively. For example, in Figure 11a, the abscissa is the SlopEn of node 1, and the ordinate is the SlopEn of node 5.

As can be observed from Figure 11, in the case of double-feature extraction, the WSO-HSlopEn distribution of sample signals belonging to the same type is relatively concentrated compared to other hierarchical entropies; for the other four types of hierarchical entropies, the bearing signals between different types are more divergent, and the entropy values of different types of bearing signals are very close.

**Figure 12:**
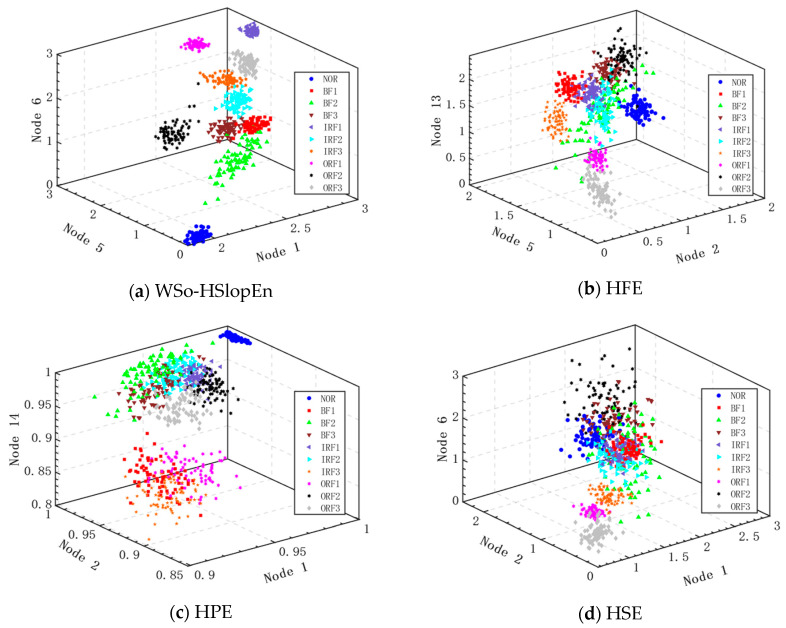

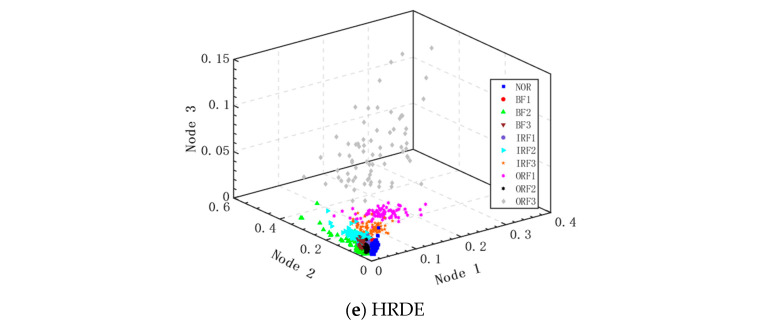
Triple-features distribution of ten types of bearing signals for different entropies.

To further improve the recognition performance, triple features were used to extract bearing fault features on various hierarchical entropies. The parameters for calculating various hierarchical entropies were the same as those of double features. Figure 12 presents the triple-feature distributions of ten types of bearing signals for different hierarchical entropies.

It can be seen from Figure 12 that there is almost no overlap based on the WSO-HSlopEn, but the feature distributions of the BF2 and IRF2 samples are relatively low in clustering; for the other hierarchical entropies, the clustering of the feature distributions of the samples are very poor because of their approximate entropy distributions. Nevertheless, the entropy distribution of WSO-HSlopEn is more dispersed, and WSO-HSlopEn of different fault types are quite different, which effectively verifies the validity of WSO-HSlopEn as a feature extraction method for ten types of bearing signals.

Next, WSO-SVM is used to construct a fault diagnosis model. The highest recognition rate is calculated for the five types of hierarchical entropies under multi-feature extraction, as shown in Table 7, where (1,5) indicates the combination of nodes with the highest recognition rate for two features are node 1 and node 5, (1,5,6) indicates the combination of nodes with the highest recognition rate for three features are node 1, node 5 and node 6, and so on.

Table 7 shows that no matter how many features are extracted, the recognition rate of these ten types of bearing signals using WSO-HSlopEn is higher than that of other hierarchical entropies; additionally, the more features we select, the better the recognition effect we obtain; in the circumstances of multi-features, the recognition rates of WSO-HSlopEn are all higher than 97.5%, yet the highest recognition rates of other hierarchical entropies are all significantly below 97.5%; for WSO-HSlopEn, when five nodes are selected, that is, choosing nodes (1,5,6,7,11), the highest recognition rate of these ten types of bearing signals reaches 100%; however, the highest recognition rate of other entropies is, respectively, 3.80%, 10.53%, 16.73%, and 4.13% lower than that of WSO-HSlopEn. Through the above comparison, we can clearly find the significant advantages of the proposed method based on WSO-HSlopEn, and the recognition results applied to diagnose faults of rolling bearings are higher than those of classic methods.

## 5. Conclusions

This paper puts forward a dual-optimization fault diagnosis method for rolling bearings based on WSO-HSlopEn and WSO-SVM. The effectiveness of the proposed methods is verified by comparing them with the classical methods. The main innovations and conclusions are as follows:

(1) On the basis of SlopEn, combined with the idea of hierarchical decomposition, HSlopEn is proposed and introduced into the feature extraction of bearing signals for the first time; at the same time, WSO is used to optimize both HSlopEn and the SVM, and WSO-HSlopEn and WSO-SVM are proposed.
(2) In the case of single-feature extraction, the proposed method based on WSO-HSlopEn has the highest recognition rate of 79.33% on node 6, which is, respectively, 19.47%, 26.67%, 31.60%, and 13.73% higher than those of HFE, HPE, HSE, and HRDE.
(3) In the case of extracting multi-features, the recognition rates are higher than 97.5%, which is a significant improvement compared with the single-feature extraction method; moreover, with the different number of features, the recognition rate based on WSO-HSlopEn is always high than the other hierarchical entropies.
(4) For the proposed dual-optimization fault diagnosis method for rolling bearings, based on WSO-HSlopEn and WSO-SVM, the more features we select, the better the recognition effect we obtain. When five nodes are selected, the highest recognition rate reaches 100%.

The proposed WSO-HSlopEn and WSO-SVM solve the problem of dependent parameter settings for SlopEn and the SVM, respectively, and their superiority has been confirmed in fault diagnosis. Therefore, WSO-HSlopEn and WSO-SVM are expected to be applied to other fields in future work, such as underwater acoustic signal processing and medical signal classification.

## Figures and Tables

**Table 1:** Fault diagnosis sample collection of bearing signals.

Bearing Condition	Defect Size (Inches)	Sample Label
NOR	-	1
IRF1	0.007	2
IRF2	0.014	3
IRF3	0.021	4
BF1	0.007	5
BF2	0.014	6
BF3	0.021	7
ORF1	0.007	8
ORF2	0.014	9
ORF3	0.021	10

**Table 2:** The recognition rate of HSlopEn for each single-feature node (nodes 1–7).

Optimization Algorithm	Recognition Rate for Each Node (%)						
	1	2	3	4	5	6	7
WSO	78.40	79.20	70.13	78.00	73.47	79.33	45.73
SO	77.47	74.80	67.87	76.32	72.27	75.43	43.28
MPA	74.00	75.87	62.93	71.20	66.93	76.53	40.40
SSA	74.67	73.07	68.53	71.87	70.67	75.60	45.07

**Table 3:** The recognition rate of HSlopEn for each single-feature node (nodes 8–14).

Optimization Algorithms	Recognition Rate for Each Node (%)						
	8	9	10	11	12	13	14
WSO	51.60	72.27	59.60	66.93	62.80	69.07	70.93
SO	48.37	70.26	58.23	65.27	60.80	68.27	69.73
MPA	39.87	70.80	56.53	60.13	56.13	67.87	65.47
SSA	50.40	69.73	57.73	63.87	60.93	68.42	69.57

**Table 4:** The highest recognition rate of HSlopEn for multi-features for four types of optimization algorithms.

Optimization Algorithms	Parameter	Number of Extracted Features			
		2	3	4	5
WSO	Highest recognition rate (%)	97.87	99.60	99.87	100
SO	Highest recognition rate (%)	87.60	90.80	93.87	96.20
MPA	Highest recognition rate (%)	67.87	84.37	86.67	89.47
SSA	Highest recognition rate (%)	64.13	70.40	74.67	83.27

**Table 5:** Recognition rates of single features for the five types of hierarchical entropies (nodes 1–7).

Entropies	Recognition Rates of Each Node (%)						
	1	2	3	4	5	6	7
WSo-HSlopEn	78.40	79.20	70.13	78.00	73.47	79.33	45.73
HFE	57.07	55.06	37.73	55.33	42.67	59.73	32.53
HPE	43.73	52.53	24.80	40.93	38.13	46.53	18.80
HSE	39.07	47.60	23.87	36.27	21.07	46.67	18.40
HRDE	55.73	65.47	33.87	53.87	42.40	54.53	30.67

**Table 6:** Recognition rates of single features for the five types of hierarchical entropies (nodes 8–14).

Entropies	Recognition Rates of Each Node (%)						
	8	9	10	11	12	13	14
WSo-HSlopEn	51.60	72.27	59.60	66.93	62.80	69.07	70.93
HFE	27.07	43.73	36.40	38.93	36.40	45.73	40.27
HPE	14.80	29.60	22.27	29.07	20.80	32.27	26.40
HSE	20.40	28.67	20.40	21.60	20.53	37.33	25.87
HRDE	25.07	45.60	37.87	42.93	37.07	50.13	48.93

**Table 7:** Highest recognition rate for the five types of hierarchical entropies for multi-features.

Entropy	Parameter	Number of Extracted Nodes			
		2	3	4	5
WSo-HSlopEn	Highest recognition rate (%)	97.87	99.60	99.87	100
	Choose the node	(1,5)	(1,5,6)	(1,5,6,7)	(1,5,6,7,11)
HFE	Highest recognition rate (%)	87.60	90.80	93.87	96.20
	Choose the node	(2,6)	(2,5,13)	(1,3,4,6)	(2,5,6,12,14)
HPE	Highest recognition rate (%)	67.87	84.37	86.67	89.47
	Choose the node	(2,4)	(1,2,14)	(2,3,5,10)	(1,5,7,12,14)
HSE	Highest recognition rate (%)	64.13	70.40	74.67	83.27
	Choose the node	(2,5)	(1,2,6)	(1,2,3,6)	(2,3,7,12,14)
HRDE	Highest recognition rate (%)	80.60	89.87	93.87	95.87
	Choose the node	(1,2)	(1,2,3)	(1,2,4,6)	(2,5,6,11,12)

## Data Availability

The data used to support the findings of this study are available from the corresponding author upon request.

## References

[1] Yan X., Jia M. (2019). Intelligent fault diagnosis of rotating machinery using improved multiscale dispersion entropy and mRMR feature selection. Knowl. Based Syst..

[2] Li X., Xu Y., Li N., Yang B., Lei Y. (2023). Remaining Useful Life Prediction with Partial Sensor Malfunctions Using Deep Adversarial Networks. IEEE CAA J. Autom. Sin..

[3] Saucedo-Dorantes J.J., Arellano-Espitia F., Delgado-Prieto M., Osornio-Rios R.A. (2021). Diagnosis Methodology Based on Deep Feature Learning for Fault Identification in Metallic, Hybrid and Ceramic Bearings. Sensors.

[4] Li Y., Tang B., Geng B., Jiao S. (2022). Fractional Order Fuzzy Dispersion Entropy and Its Application in Bearing Fault Diagnosis. Fractal Fract..

[5] Li X., Yu S., Lei Y., Li N., Yang B. (2023). Intelligent Machinery Fault Diagnosis with Event-Based Camera. IEEE Trans. Ind. Inform..

[6] Yadav E., Chawla V.K. (2022). An explicit literature review on bearing materials and their defect detection techniques. Mater. Today Proc..

[7] Hu Q., Li Y., Sun X., Chen M., Bu Q., Gong B. (2023). Integrating test device and method for creep failure and ultrasonic response of methane hydrate-bearing sediments. Rev. Sci. Instrum..

[8] Li Y., Tang B., Jiang X., Yi Y. (2022). Bearing Fault Feature Extraction Method Based on GA-VMD and Center Frequency. Math. Probl. Eng..

[9] Li Y., Jiao S., Geng B. (2023). Refined composite multiscale fluctuation-based dispersion Lempel–Ziv complexity for signal analysis. ISA Trans..

[10] Li Y., Geng B., Tang B. (2023). Simplified coded dispersion entropy: A nonlinear metric for signal analysis. Nonlinear Dyn..

[11] Richman J.S., Moorman J.R. (2000). Physiological time-series analysis using approximate entropy and sample entropy. Am. J. Physiol. Heart Circ. Physiol..

[12] Bandt C., Pompe B. (2002). Permutation entropy: A natural complexity measure for time series. Phys. Rev. Lett..

[13] Rostaghi M., Azami H. (2016). Dispersion Entropy: A Measure for Time Series Analysis. IEEE Signal Process. Lett..

[14] Han M., Pan J. (2015). A fault diagnosis method combined with LMD, sample entropy and energy ratio for roller bearings. Measurement.

[15] Xue X., Li C., Cao S., Sun J., Liu L. (2019). Fault Diagnosis of Rolling Element Bearings with a Two-Step Scheme Based on Permutation Entropy and Random Forests. Entropy.

[16] Dhandapani R., Mitiche I., McMeekin S., Mallela V.S., Morison G. (2021). Enhanced Partial Discharge Signal Denoising Using Dispersion Entropy Optimized Variational Mode Decomposition. Entropy.

[17] Cuesta-Frau D. (2019). Slope Entropy: A New Time Series Complexity Estimator Based on Both Symbolic Patterns and Amplitude Information. Entropy.

[18] Li Y., Tang B., Jiao S. (2023). SO-slope entropy coupled with SVMD: A novel adaptive feature extraction method for ship-radiated noise. Ocean Eng..

[19] Li Y., Tang B., Yi Y. (2022). A novel complexity-based mode feature representation for feature extraction of ship-radiated noise using VMD and slope entropy. Appl. Acoust..

[20] Cuesta-Frau D., Dakappa P.H., Mahabala C., Gupta A.R. (2020). Fever Time Series Analysis Using Slope Entropy. Application to Early Unobtrusive Differential Diagnosis. Entropy.

[21] Cuesta-Frau D., Schneider J., Bakštein E., Vostatek P., Spaniel F., Novák D. (2020). Classification of Actigraphy Records from Bipolar Disorder Patients Using Slope Entropy: A Feasibility Study. Entropy.

[22] Shi E. (2022). Single Feature Extraction Method of Bearing Fault Signals Based on Slope Entropy. Shock. Vib..

[23] Jiang Y., Peng C.K., Xu Y. (2011). Hierarchical entropy analysis for biological signals. J. Comput. Appl. Math..

[24] Xing J., Xu J. (2022). An Improved Incipient Fault Diagnosis Method of Bearing Damage Based on Hierarchical Multi-Scale Reverse Dispersion Entropy. Entropy.

[25] Peng C., Zhao X., Jiang H. (2021). A New Method of Fault Feature Extraction Based on Hierarchical Dispersion Entropy. Shock. Vib..

[26] Wang X., Si S., Li Y. (2020). An integrated method based on refined composite multivariate hierarchical permutation entropy and random forest and its application in rotating machinery. J. Vib. Control.

[27] Xue Q., Xu B., He C., Liu F. (2021). Feature Extraction Using Hierarchical Dispersion Entropy for Rolling Bearing Fault Diagnosis. IEEE Trans. Instrum. Meas..

[28] Keller J.M., Gray M.R., Givens J.A. (1985). A fuzzy K-nearest neighbor algorithm. IEEE Trans. Syst. Man Cybern..

[29] Svetnik V., Liaw A., Tong C., Culberson J.C., Sheridan R.P., Feuston B.P. (2003). Random Forest:  A Classification and Regression Tool for Compound Classification and QSAR Modeling. J. Chem. Inf. Comput. Sci..

[30] Samanta B., Al-Balushi K.R., Al-Araimi S.A. (2003). Artificial neural networks and support vector machines with genetic algorithm for bearing fault detection. Eng. Appl. Artif. Intell..

[31] Wang Z., Yao L., Cai Y., Zhang J. (2020). Mahalanobis semi-supervised mapping and beetle antennae search based support vector machine for wind turbine rolling bearings fault diagnosis. Renew. Energy.

[32] Tang H., Yuan Z., Dai H., Du Y. (2020). Fault Diagnosis of Rolling Bearing Based on Probability box Theory and GA-SVM. IEEE Access.

[33] Ye M., Yan X., Jia M. (2021). Rolling Bearing Fault Diagnosis Based on VMD-MPE and PSO-SVM. Entropy.

[34] Jin Z., Chen G., Yang Z. (2022). Rolling Bearing Fault Diagnosis Based on WOA-VMD-MPE and MPSO-LSSVM. Entropy.

[35] Braik M., Hammouri A., Atwan J. (2022). White Shark Optimizer: A novel bioinspired meta-heuristic algorithm for global optimization problems. Knowl. Based Syst..

[36] Case Western Reserve University Bearing Data Center. https://engineering.case.edu/bearingdatacenter.

[37] Fatma A.H., Abdelazim G.H. (2022). Snake Optimizer: A novel meta-heuristic optimization algorithm. Knowl. Based Syst..

[38] Afshin F., Mohammad H., Seyedali M., Amir H.G. (2020). Marine Predators Algorithm: A nature-inspired metaheuristic. Expert Syst. Appl..

[39] Xue J., Shen B. (2020). A novel swarm intelligence optimization approach: Sparrow search algorithm. Syst. Sci. Control Eng..

